# Socio-cultural and economic determinants of Latrine ownership and utilisation: a community-based survey in Bole district of Ghana

**DOI:** 10.1186/s12889-024-20521-w

**Published:** 2024-10-31

**Authors:** Jonathan K. Nanyim, Abdul-Manaf Mutaru, Collins Gbeti, Abdul Rahaman Issahaku, Abdulai Abubakari, Abukari Wumbei

**Affiliations:** 1 Department of Behavioural Sciences, School of Public Health, University for Development Studies, Tamale, Ghana; 2 School of Engineering, University for Development Studies, Tamale, Ghana; 3 Department of Global and International Health, School of Public Health, University for Development Studies, Tamale, Ghana; 4 Drylands Research Institute, University for Development Studies, Tamale, Ghana

**Keywords:** Community-Led Total Sanitation, Economic, Latrine Ownership, Open Defecation, Socio-Cultural, and Utilisation

## Abstract

Latrine ownership and utilization is an integral part of the Community-Led Total Sanitation concept. This study assessed ownership and utilization of latrines in selected Open Defecation and Open Defecation-Free communities within the Bole district. A cross-sectional survey design and quantitative approach were employed in this study. Simple random sampling was used to select 166 households from 15 Open Defecation and 5 Open Defecation-Free communities. Data collection was done using a structured questionnaire. Data was analyzed using descriptive statistics, chi-square, and binary logistic regression. The proportion of latrine ownership and utilization were 22.3% and 6.6% respectively. Educational qualification, tertiary education (aOR: 6.1; 95% CI: 1.05–35.56), household subjective norms (OR: 0.19; 95% CI: 0.04–1.01), and awareness of Community-Led Total Sanitation (aOR: 13.3; 95% CI: 2.95–60.24) were determinants of latrine ownership and or utilisation. In conclusion, latrine ownership and use were generally low with education, awareness of community-led total sanitation, residential status, and household subjective norms as factors associated with ownership and or utilization. Education or sensitization should target cultural norms impeding latrine construction and usage. Again, implementable by-laws/community regulations must be implemented to propel latrine ownership and utilization.

## Introduction

Latrine ownership and utilisation is an integral part of the Community-Led Total Sanitation (CLTS) implementation. The major aim of the CLTS concept is to end open defecation by moving people from the practice of open defecation (OD) and the “dig and bury” system to the ownership and use of improved latrines [1]. Even though the focus of the CLTS concept is not on latrine construction, its ownership and utilisation are extremely crucial for the success and sustainability of the entire CLTS concept [1].

Over the years, latrine ownership has been low worldwide [2], which means that a substantial number of people in the world may be resorting to open defecation other than the use of household latrines. Although worldwide coverage for safely managed sanitation services has increased from 47.1% in 2015 to 54% in 2020, there are as high as 3.6 billion people in the world who do not have access to improved toilet facilities including 1.7 billion who are currently practicing open defecation with its accompanying negative externalities [3, 4]. The situation is not different in Sub-Saharan Africa, as about 779 million people do not have access to basic sanitation facilities including 208 million people who do not have access to toilet facilities and are currently engaged in OD practice and 839 million who lack access to basic hygiene services [3].

In Ghana, the number of people practicing open defecation was reported at 18% in 2020, which translates into a little over 5 million people engaged in the act [5, 6]. The practice is most prevalent in the Upper East Region with about 89% of the people without any form of latrine, followed by the Northern and Savannah Regions with about 72%, and the Upper West Region with about 71% [7]. It is estimated that 8 out of every 10 Ghanaians within the rural settings are without toilet facilities and are engaged in one form of open defecation or another, of which the Savannah Region is not an exception [8].

Latrine ownership and utilisation in Bole have been generally low. For instance, a baseline survey conducted by the Swiss Federal Institute of Aquatic Science and Technology (eawag) in 2016 revealed that Bole district has 97% of its population engaging in open defecation, which means that only about 3% of the population of the district had access to toilet facilities as at the time [9]. Even though the situation of having access to toilet facilities has improved from the previous 3% in 2017 to 30.8% in 2019 and reduced OD from 97 to 69.2% due to the ongoing CLTS activities by NGOs in the WASH sector in the district, the situation remains unsatisfactory [10, 11].

The reason for the low adoption and utilisation of toilet facilities and the wide practice of open defecation is a question that many have attempted but has remained largely unanswered. The situation as literature [6, 12, 13] has it, is often connected with some identified socio-cultural and economic factors. One of the greatest hindrances in accepting any sanitation system has to do with the financial cost associated with it. Osumanu and Kosoe in 2019 intimated that how well a group of people receives a particular sanitation system and how technically feasible that system is, is largely dependent on several factors including cost, affordability as well as communal or household characteristics [6]. Therefore, there is a relationship between household wealth and the ownership of an improved sanitation system such as a latrine as well as the practice of open defecation [6]. Also, lessons learned from introducing microfinance loans for sanitation in rural Cambodia revealed a huge increase in sanitation uptake among the poor, suggesting that a small loan size and a poor-inclusive application process are essential for the success of any sanitation system [14]. Additionally, the type of job and the occupation one is engaged in also has a lot of impact on sanitation success as residents of Nadowli-Kaleo believed secured jobs with regular incomes can motivate them to construct and maintain latrines [15].

According to Jenkins and Scott, 2007, the adoption of latrines in poor communities follows three behavioural patterns: preference, intention, and choice [16]. Social norms are usually the rules that govern how a group of people in a community behaves. This includes the behaviour that exists in the community for people to conform or otherwise [17, 18]. Therefore, the presence or absence of these social norms, are significant contributors to the adoption and use of household latrines [17, 19]. For instance, peers, family members, and others in the community will ignore the use of a latrine and practice OD, if that is a common norm in the community rooted in culture and tradition and learned from childhood and vice versa [17, 19]. Evidence from several studies has pointed out that human behaviour is not only controlled by attitudes [20], perceived behavioural control [21], perceived barriers [22], and risk assessments [23] but also by perceptions about others’ beliefs [24] and behaviours [25]. This is what overall, makes up individuals/households’ subjective norms. In Peru, open defecation is held as “the most natural thing,” as observed by Connell, he explained that the practice is traditional, habitual, and part of one’s daily routine, and as such these social norms are also held strongly by those who practice them and, in this case, open defecators [17].

In Uganda, it was reported that in the late 1940s, people were afraid to use latrines in the sense that their fixed location would provide sorcerers with easy access to their excreta for devilish purposes. In the same vein, someone’s faeces was not allowed to come into contact with another for fear of “Spiritual Contamination,” therefore, defecating in the bush and the surroundings was considered a safer option [26]. Cultures in the Western Region of Ghana have forbidden women undergoing menstruation from sharing toilet facilities with others, especially the males, with the belief that they are unclean during this period [17]. Likewise, in Madagascar, it is taboo to store sewerage underground for fear of contaminating the dead [27, 28]. Similar superstitious and traditional beliefs have been reported by [29] from Tamale (Ghana). Also [18] and [30], made similar observations in communities in Burkina Faso, Mali, and for the Idoma people in Nigeria, and in Wa (Ghana) respectively. The high open defecation rate in Bole District [10, 11] despite the ongoing CLTS implementation efforts by government and non-governmental has necessitated the current study that examined the sociocultural and economic factors influencing the ownership and utilization of household latrines in Bole-District in the Savannah Region of Ghana.

## Materials and methods

### Study area and description

The study was conducted in the Bole District of the Savannah Region, Ghana. The Bole district was created by Legislative Instrument (L.I) 1786 in 2004 and is situated between latitudes 8’10.5 and 09’ and longitudes 1.50E’ and 2.45 W in the Northern/Savanah area. The district is around 4,800 square kilometres in size [7]. The population of the district at the time of this study is 61,593 with 31,022 and 30,571 being Males and Females respectively. The area is predominantly agrarian with pockets of trading and small-scale mining activities. The people mostly practice a centralized traditional political system headed by a paramount chief in Bole. The Major tribes in the area include Gonja, Safalba, Vagla, Mo, and the Brifo who are mostly migrant settlers. The district has just one Small Town Water System which is located at the district capital and a few boreholes across the communities. The district has poor drainage systems with the majority of its citizens engaged in open defecation practices [7].

### Study design

This was a community-based cross-sectional survey with a quantitative approach. The study was conducted among households drawn from open defecation communities (OD) communities in the Bole District, Ghana.

### Population and sampling

The population for this study constituted non-ODF communities in the district. However, households without latrines in ODF communities and households in OD communities were involved in determining the factors influencing non-ownership and utilization of latrines as required by the CLTS concept. That is, the process aimed at ending open defecation and encouraging the use of simple household latrines. Once a community goes through downs steps to get simple household latrine constructed and used, CLTS concept is implemented.

According to the Bole district annual report for 2019, there were 190 communities in the district. Records from Global Communities Ghana, an international NGO spearheading the implementation of CLTS in the district, and the district environmental health unit, the majority (104) communities were OD, 5 of the communities were at the end stage for ODF but had not been declared ODF per CLTS implementation protocols. The remaining 86 had been declared ODF before data collection for this study in March 2020 [31]. With the above data, a total of 20 communities were selected. The process of selection of communities was simply random while verifying their actual status as OD.

From the district assembly’s records, 296 households within the 20 communities were selected. Based on this number of households, the Krejcie and Morgan table for sample size determination [32] was adopted in sampling households. A total of 166 households were randomly sampled. To ensure equal representation, the sample size was further distributed proportionally to the 20 communities based on their respective household population. The household size of a community was divided by the determined sample size (166) multiplied by the 20 communities. On average, 8 households were interviewed for the study.

### Inclusion and exclusion criteria

To be eligible to participate in the study, a household head or representative must be 18 years of age or older, and the household must also be involved in the CLTS initiative. This was to ensure that only households involved in the CLTS initiative in the communities, more precisely, latrine ownership and utilization, were those engaged for the needed information.

### Data collection tools and techniques

Data was collected using a questionnaire. The questionnaire was adopted from a related study [13] based on a careful review of the literature. The questionnaire was drafted into three main themes or sections, that is (i) demographic indicators, (ii) latrine ownership and utilization, and (iii) factors influencing latrine ownership and utilization.

The questionnaire was designed in the English language, but with the assistance of the WASH officer and district environmental health officer at the Bole district assembly, the questionnaire was first interpreted in the local language for a clearer understanding of its content and how the questions could be asked to obtain the desired responses needed, especially for respondents (household heads) who could not read or comprehend the English language.

### Validity and reliability

After getting the translation of the questionnaire into the local language, the questionnaire was piloted among 20 households in three OD and ODF communities in a sister district (Sawla Tunna Kalba district). The content of the questionnaire was modified with all required corrections made after the pilot study. Both the face and content validity were performed by two independent persons (WASH and environmental health specialists) in both districts (Bole and Sawla Tunna Kalba). The reliability and internal consistency of the instrument were measured using Cronbach’s alpha coefficient of reliability. The alpha value for scales on factors influencing household latrine use was 0.71 which is considered acceptable (33–34).

### Data analysis

Microsoft Office (Excel 2016) and SPSS version (v25) were the main software used for data processing and analysis respectively. After data cleaning and audit, demographic and latrine ownership variables were analyzed descriptively using frequencies. The factors (household subjective norms, cultural factors, and economic factors) were assessed on a five-point Likert scale (strongly agree-1, agree-2, neutral-3, disagree-4, and strongly disagree-5). The mean and standard deviation for each of the variables under each factor were determined to precisely determine the direction of the responses. In this regard, a mean score or rating of less than 3 for a variable indicated agreeable behaviour, while a mean score rating greater than 3 was described as disagreeable behaviour for all variables [13].

To obtain the overall agreeable and disagreeable behaviour of respondents for the three main factors (household subjective norms, cultural, and economic factors), composite means were determined for each variable under each of the factors, and a cumulative mean was then generated for all variables considered. The composite means were then compared to the cumulative mean and a composite mean that was less than the cumulative mean was coded as 1, while any composite means greater than the cumulative mean was coded as 2 (1-agreed behaviour and 2-disagreed behaviour). These codes were used to generate and determine a binary response (overall agreed or disagreed behaviour) for the respective factors (household subjective norms, cultural, and economic).

A Pearson’s chi-square association and binary logistic regression analysis were further conducted to determine an association between dependent variables (latrine ownership and utilization) and independent variables (demographic variables, factors, and awareness of the CLTS program). A precision level of 0.05 was set for bivariate (Chi-square) analysis. Further, a multivariate binary logistic regression analysis was executed at a 95% confidence level. Data collection cleaning and analyses was four [5] months, beginning December 2022 to March 2023.

### Ethical consideration

The study sought research approval from the Committee of Human Research and Publications Ethics (CHRPE/AP/645/23) of the Kwame Nkrumah University of Science and Technology, Kumasi, Ghana. Again, permission was obtained from the Bole District Assembly (the environmental health and sanitation division). The consent of community opinion leaders (chiefs and assemblymen) in selected communities was also obtained.

When a participant was not formally educated, a legal guardian provided consent on behalf of such participants. This was after the content of the study’s information sheet, containing the purpose of the study and consent form, was read in English, clarified, and translated into the local language (Gonja). This was done in the presence of both the legal guardian and the participant to ensure the best understanding of the participant. All COVID-19 protocols such as social distancing, wearing of nose masks, and use of hand sanitizers as outlined by the Ministry of Health (MoH) and Ghana Health Service (GHS) were adopted due to the personal interactive nature of the data collection process.

## Results

### Socio-demographics characteristics

Table 1 shows that 166 households were enrolled with a 100% response rate. The majority of participants (65.7%) identified as males while 34.3% were females. Less than half (41.6%) were either 35 years of age or younger with an average age of 42.6 ± 15.7 years. Half of the participants had not acquired a formal education. Approximately 43.4% were Muslims and the majority (39.8%) were Gonjas, the predominant ethnic group in the Bole district and the Savannah region. More than half (62.0%) of participants were living in rural communities.

**Table 1:** Socio-demographics characteristics (*N* = 166)

Variables	Frequency	Percentage
**Sex**		
Male	109	65.7
Female	57	34.3
**Age (years)**		
≤ 35	69	41.6
36–52	56	33.7
53–69	27	16.3
70–85	14	8.4
Mean (SD)	42.6 ± 15.7	
**Education Qualification**		
No Formal	84	50.6
Primary school education	63	38.0
Middle/SHS	11	6.6
Tertiary	8	4.8
**Religion**		
Christian	44	26.5
Muslim	72	43.4
Traditional	50	30.1
**Ethnic group**		
Gonja	66	39.8
Brifo	32	19.3
Vagala	11	6.6
Others	57	34.3
**Residential Setting**		
Rural	103	62.0
Urban	63	38.0

SD; Standard Deviation

### Ownership of Latrine and utilisation

Table 2 indicates that in this current study, more than three-quarters (77.7%) of households did not own a household latrine. Also, the majority (93.4%) reported to have been practicing open defecation.

**Table 2:** Latrine ownership and utilization (*N* = 166)

Variables	Frequency	Percentage
**Ownership of latrine and hand washing facility**		
Yes	37	22.3
No	129	77.7
**Latrine utilisation**		
Household latrine	11	6.6
Open Defecation	155	93.4

### Perceived factors influencing Latrine ownership

From Table 3, results for factors influencing latrine ownership show that, respondents generally agree to household subjective norms or social factors, as evident in mean responses to the questions or variables in Table 3. The mean rating/score (x̅) for the statement “defecating openly because people who matter to me in society do so” was 2.95, and the standard deviation (SD) of 1.54, indicating an agreed behaviour to the statement. Similarly, “defecating openly because all the people around me do same” recorded a mean rating and standard deviation of 2.83 ± 1.55, an indication of agreed behaviour to the statement. A mean rating of 3.89 ± 1.11 shows respondents expressed disagreement with the statement “People who are important to me think that I should defecate in the open”.

**Table 3:** Perceived factors influencing household latrine ownership

Variables	x̅	SD
**Household Subjective Norms**		
People who are important to me think that I should defecate in the open	3.89	1.11
Defecating open because people who matter in society do so	2.95	1.54
Defecating open because all the people around me do the same	2.83	1.55
Defecating open because I feel it is normal to do so in my community	2.80	1.57
**Cultural Factors**		
Faeces must always be kept far away from the house and must not be kept in any room closer to or within the house	2.21	1.47
One can be possessed by evil spirits by defecating in a toilet	3.16	1.42
Men’s and women’s faeces should not mix, therefore the need for OD	3.63	1.40
Continuous defecation in a toilet weakens your magical powers	3.16	1.33
Defecating in an enclosed place inside the toilet is a taboo	3.81	0.94
Children can defecate in the open as their faeces are considered harmless	2.40	1.41
Practicing OD signifies the continuation of ancestors’ way of life	2.01	1.39
Menstruating women are not supposed to defecate in a toilet	3.34	1.41
Shared toilets are usually associated with evil spirits	2.95	1.44
**Economic factors**		
Inability to construct a latrine because of lack of job	2.81	1.67
Inability to hire labour to construct one due to financial constraints	2.23	1.51
Lack of support from the extended family to help own a latrine	2.61	1.56
Inability to own a latrine because of the cost of construction materials e.g., cement, iron rods, etc.	2.20	1.55
Instituting Village Savings Groups in this community to provide support for households will help accelerate CLTS activities	2.46	1.64
Availability of financial support can aid in the construction and owning of a latrine	2.31	1.55
Willingness to stop OD, if the agriculture sector becomes lucrative	2.44	1.59

Note: x̅= mean rating, SD = standard deviation, x̅ < 3.00 = agree behaviour. x̅ > 3.00 = disagree behaviour, OD = Open Defecation

For cultural factors influencing latrine ownership, the results revealed a mixed response to the various statements outlined. The majority of the participants (x̅=2.2 ± 1.47) agreed with the statement “feces must always be kept far away from the house and must not be kept in any room closer to or within the house”. Most of the respondents disagreed with the assertion that “one can be possessed by evil spirits by defecating in a toilet”, mean, 3.16 ± 1.42.

### Factors associated with Larine ownership and utilisation and determinants of Larine ownership and utilisation

#### Latrine ownership

As indicated in Table 4, in this study a Pearson’s chi-square analysis showed education qualification (***X***^***2***^ = 10.43, *p* < 0.05) and awareness of CLTS (***X***^***2***^ = 17.09, *p* < 0.0001) to be statistically significantly associated with latrine ownership. A multivariate binary logistic regression analysis revealed that educational qualification is significantly associated with latrine ownership. Those who have acquired tertiary education were 6 times more likely to own a household latrine compared to those without formal education (aOR: 6.1; CI: 1.05–35.56; *p* ≤ 0.20). Awareness of CLTS (aOR: 13.3; CI: 2.95–60.24; ) was also significantly associated with latrine ownership. Those who had heard about CLTS were 13 times more likely to own a household latrine than those who never heard of CLTS.

**Table 4:** Factors associated, with and determinants of latrine ownership and utilisation (*N* = 166)

Variable	Latrine ownership				Latrine Utilisation			
	Yes (37)	No (129)	X^2^	aOR (95% CI)	HL (11)	OD (155)	X^2^	aOR (95%CI)
**Education Qualification**			10.43**				9.360**	
No Formal Education^1^	21 (25.0)	63 (75.0)			2 (2.4)	82 (97.6)		
Primary school education	10 (15.9)	53 (84.1)			5 (7.9)	58 (92.1)		2.53 (0.46, 14.02)
Middle/SHS	1 (9.1)	10 (90.9)			2 (18.2)	9 (81.8)		3.93 (0.42, 36.48)
Tertiary	5 (62.5)	3 (37.5)		6.1 (1.05, 35.56) **	2 (25.0)	6 (75.0)		6.83 (0.65, 71.47)
**Residential Setting**			0.135				3.300*	
Rural ^1^	22 (21.4)	81 (78.6)			4 (3.9)	99 (96.1)		
Urban	15 (23.8)	48 (76.2)			7 (11.1)	56 (88.9)		3.09 (0.76, 12.60)
**Household subjective norms**			0.096				5.335**	
Disagree^1^	17 (21.3)	63 (78.8)			9 (11.2)	71 (88.8)		
Agree	20 (23.3)	66 (76.7)			2 (2.3)	84 (97.7)		0.19 (0.04, 1.01) **
**Cultural factors**			0.100				0.005	
Disagree^1^	24 (23.1)	80 (76.9)			7 (6.7)	97 (93.3)		
Agree	13 (21.0)	49 (79.0)			4 (6.5)	58 (93.5)		
**Economic factors**			< 0.001				0.148	
Disagree^1^	12 (22.2)	42 (77.8)			3 (5.6)	51 (94.4)		
Agree	25 (22.3)	87 (77.7)			8 (7.1)	104 (92.9)		
**Awareness of CLTS**			17.09***				7.24	
Yes	35 (68.2)	75 (58.1)		13.3 (2.95, 60.24) ***	6 (5.5)	104 (94.5)		
No^1^	2 (3.6)	54 (96.4)			5 (8.9)	51 (91.1)		
**Latrine ownership**							0.169	
Yes					3 (8.1)	34 (91.9)		
No^1^					8 (6.2)	121 (93.8)		

1_ference category; *_ *p* < 0.20; **_ *p* ≤ 0.05; ***_ *p* ≤ 0.001; HL_Household Latrine; OD_Open Defecation; aOR Adjusted Odds Ratio

#### Latrine utilisation

From Table 4, a Pearson’s chi-square association test indicated education qualification (***X***^***2***^ = 9.36, *p* < 0.05), residential setting (***X***^***2***^ = 3.30, *p* < 0.20), and household subjective norms (***X***^***2***^ = 5.34, *p* < 0.05) to be significantly associated with the utilisation of household latrine. After adjusting for confounders, a multivariate binary logistic regression showed household subjective norms to be statistically significant. Those who subscribe to the household subjective norms were significantly 0.19 times less likely to utilize a household latrine than those who do not subscribe (aOR: 0.19; CI: 0.34–1.01; *p* ≤ 0.20).

## Discussion

The study revealed that there is a very low level of latrine ownership and utilisation among households in the Bole district. What is revealing about this result is that whereas ownership was at 22.3% among respondents, utilisation was at 6.6%. This means that even among households who own latrines some do not use it. This finding is consistent with what [13] and [12] when they recorded high open defecation and low latrine utilisation rates among CLTS beneficiaries and school pupils in Savannah and Volta regions of Ghana respectively. It, however, differs from Harter’s study which found improvement in latrine utilisation and a reduction of open defecation as a result of CLTS intervention [35]. This result as obtained could also be explained by the fact that most of the study communities were communities in which the CLTS process was still ongoing and therefore since they have not attained the open defecation-free (ODF) status, latrine ownership and utilisation were expected to be low.

This study has found that human behaviour is controlled by individuals’/households’ subjective norms except for role model influence which is consistent with the literature that human behaviour is not only controlled by attitudes [25], perceived behavioural control [21], perceived barriers [22], and risk assessments [23] but also by perceptions about others’ beliefs [24] and behaviours [25]. Therefore, what these pieces of literature suggest is that households’ practice of OD may have some strong fundamental roots extending from subjective norm/belief systems which include; perception about others’ beliefs-descriptive norms, role model influence, and influence by the environment. On the role model influence, the finding differs from [12] where he found a highly positive effect of descriptive norms, role models, and environmental influences on pupil’s intention to engage in OD in some parts of Volta and Western Regions of Ghana. It is also consistent with the works of [16, 17, 19] as they also found similar results.

In terms of cultural factors, this study revealed that the majority of the study participants believed that faeces and latrine facilities should not be closer to the household environment and should always be situated an appropriate distance from the house. No other relevant studies produced a similar finding, making this a new finding. This finding may imply that households will not be willing to own household latrines, and should they agree to construct latrines using the CLTS concept, they might do so far away from their houses. This may create the possibility of practicing OD, especially amongst children, during the night, or during emergencies such as diarrheal illnesses. This study reported that participants disagreed that an individual could be possessed by evil spirits for defecating in a toilet. This result is contrary to what previous studies have found in Sierra Leone [36], Kampala [37], and Ghana [6, 38] where people avoid the use of latrine facilities for fear of becoming possessed or hunted by evil spirits. The current results could be attributed to the ongoing CLTS activities in the Bole District where households are being given education on latrine ownership and utilisation. Another finding made by this paper is that men and women can have their faeces mixed by using the same latrine facility. This result is different from what Mukherjee et al. found among ethnic cultures in South Asia particularly in Nepal, where father-in-law and daughter-in-law cannot use the same latrine facility and also forbid women undergoing menstruation from sharing latrine facilities with others, especially the males with the belief that they are unclean during this period [39]. This outcome could be attributed to the efforts of NGOs implementing CLTS and Menstrual hygiene Activities in the District. Reports from the residents of Wa East indicate that latrine usage will weaken one’s magical powers [40]. This study, however, revealed that latrine usage does not influence one’s magical power. The variations in the results can be attributed to the differences in cultures and also the ongoing CLTS activities by implementing partners in the district. The people are believed to be better informed by the educational efforts of these organizations and hence the outcome.

Taboos and superstitions surrounding human faeces are key socio-economic and physical factors influencing latrine ownership and utilisation. For instance, in Madagascar, it is taboo to store sewerage underground for fear of contaminating the dead. In the same vein, one person’s faeces cannot be put on top of another person’s own, both of which exclude the use of drop and store systems [27, 28]. In Uganda, it is forbidden to use cesspits because it is believed that sorcerers can use the excreta to inflict pain on individuals. Similar studies among the Idoma communities in Nigeria also revealed that it is taboo to use a latrine, and many older people still do not defecate in enclosed places [26, 29]. However, this study has revealed that it is not a taboo to defecate in a latrine facility as was found in other jurisdictions. The differences in the findings may result from cultural evolution over the years and perhaps due to education.

Traditionally, most people have considered children’s faeces as harmless. In this study majority of the respondents, agree that children can continue to defecate in the open since their faeces are considered as harmless. The result is consistent with findings from Harter’s study in rural communities in Northern Ghana [35] where they reported that the faeces of children greater than 3 years are more likely to be properly disposed of than those less than 3 years even though there was access to on-site latrines [41]. The result is also consistent with [42, 43] which found unsafe disposal of child faeces in rural India, Ethiopia, and South Asia where young children’s faeces are considered less harmful and less disgusting than older children’s faeces [44].

This study has further found that the majority of the participants believe they practice open defecation because it is a way of continuing their ancestors’ way of life. They believe it is a way of protecting the ancestral legacy and also showing respect to the ancestors. This result is consistent with what WaterAid found among the tribes of the Malian and the Idoma people of Nigeria where OD is viewed as an ancestral practice passed down through generations [29]. Similar findings from researchers in India indicate that OD is a long-standing habit, more comfortable, and provides an opportunity to enjoy fresh air [45]. This study reveals that though one cannot be possessed by evil spirits by just using a household toilet, however, they believed shared toilets (public toilets) are usually associated with evil spirits and therefore, using such toilets will lead to one being possessed by evil spirits. This finding is consistent with what WaterAid found among the residents of Wa East and Tamale Metropolis [40]. This finding is positive and can promote latrine ownership and utilisation at the household level.

According to Minh & Hung 2011, while unimproved sanitation comes with a lot of economic consequences to populations and nations, improved sanitation also comes with huge financial costs in terms of investment and recurrent cost [46]. Evidence from previous studies suggests that households are unable to embrace any sanitation systems because of the economic implications associated with it. For instance, residents of Nadowli-Kaleo in Ghana believe that if they have secured jobs with regular incomes it can motivate them to construct and maintain latrines, and that will lead to sustained improved sanitation behaviour [15]. In the same light [6], revealed that the type of occupation or economic activity one is engaged in influences his/her OD decision. The current findings revealed that the majority of the study participants believed that they were unable to construct latrines to end open defecation because they had no jobs. This finding underscores the need for CLTS implementation to collaborate with job-creating sectors such as the agricultural sector, which is readily available for rural dwellers. It also means policymakers such as the Government must focus on building the capacity of individuals to engage in productive activities if it must succeed in its sanitation fight.

The study also revealed that the majority of the respondents believed that they are financially constrained and therefore, are unable to hire labour to construct latrines. They believed that this situation makes them continue to practice OD even though CLTS is being implemented in their communities. This finding confirms the research of [6, 38] where they found that one of the major causes of OD in Wa municipality was the fact that the majority of the respondents believed they were financially constrained and therefore could not afford construction materials or hire labour to construct latrines.

Lessons from Cambodia have indicated that instituting microfinance loans will lead to an increase in sanitation uptake especially among the poor, suggesting that a small loan size and a poor inclusive application process are essential to the success of any sanitation system [14]. Comparable to this finding, this study has also revealed that instituting Village Savings Groups in communities will help accelerate CLTS activities. They believe the move will enable them to have access to financial support in the form of loans, which will promote latrine construction and utilisation.

The study revealed that the participants believed that if they had support from the extended family, they would have been able to construct latrines and therefore end open defecation as expected of the CLTS concept. This indicates that the extended family support systems within communities have changed from how they used to work previously and therefore cannot be relied upon by the CLTS approach in its attempt to end open defecation and promote latrine construction and utilisation. This confirms the work of [6] who also found an extended family support system as one way to end open defecation.

This study has also found that if the agriculture sector is made lucrative, households will be able to own and use household latrines. They believed that this intervention would provide them with the needed jobs and financial support to own latrines. This result is consistent with other findings in Ghana [14].

### Factors associated with Latrine ownership and utilisation

Education and awareness of CLTS were found to be significantly associated with household latrine ownership. A binary logistic regression analysis conducted revealed that respondents who had attained tertiary education were approximately 6 times more likely to own a latrine as compared to those without any form of formal education. This finding corroborates that of O’Connell, 2014 who found that educated household heads were more likely to be informed better about the health consequences associated with unimproved toilet facilities and open defection and therefore, will resort to owning an improved latrine [17]. It also confirms the works of [47–49] which concluded that there is a positive association between the educational attainment of the head of a household and the probability of having access to or owning an improved latrine facility. Likewise, those who were aware of the ongoing CLTS process in the Bole district were approximately 13 times more likely to own a latrine as compared to those who were not aware of the ongoing CLTS process. This finding corroborates the work of [50], where they observed that having the right knowledge of behaviour enhances one’s intention toward the performance of that behavior and may be one of the reasons why [1] have proposed broad-based community participation during the CLTS implementation process, as broader awareness gives a better chance of a greater result in terms of latrine ownership, utilisation, and ODF attainment.

At the utilisation level, education, residential setting, and household subjective norms were found to be significantly associated with latrine utilisation. Even though not a significant predictor, those who have attained tertiary education were found to be approximately 7 times more likely to utilise their latrines. Similarly, those who have attained SHS education were also found to be approximately 4 times more likely to utilise a latrine. This finding confirms the works of [17, 51] when they investigated the determinants of ownership of toilet facilities among households. In terms of residential settings, those who are peri-urban dwellers were found to be approximately 3 times more likely to utilise a latrine as compared to their rural counterparts. This may be so because those who are in the rural areas are closer to the bush than those who are in the urban areas and therefore, they are motivated to practice OD as compared to their urban counterparts who do not have that opportunity. To add to this, the outcome may also suggest that traditional norms about latrine utilisation and OD practice are stronger in the rural areas as compared to the peri-urban and urban centers and hence the outcome. This result confirms the works of [17, 33, 48].

In addition, those who subscribed to household subjective norms as outlined by this study were found to be less likely to utilise a latrine as compared to those who did not. This finding confirms that of [12] who investigated and reported on the behavioural and cultural factors influencing OD among pupils in some parts of the Western and Volta Regions of Ghana.

## Conclusions

Findings from this study show that open defecation is still a major social problem within the Bole district despite the efforts of NGOs and other stakeholders end the practice. This is evident by the very low latrine ownership and utilization among study respondents.

Education and awareness of CLTS were found to be significantly associated with household latrine ownership and utilization, with more educated people more likely to own and use latrines more than those who are less educated or not educated.

It is recommended that more aggressive and effective measures including community-initiated regulations to deter people from practicing open defecation, which will in turn compel them to construct and utilize household or community latrines. Education and sensitization programs must be frequently organized to erode long-existed cultural and social beliefs such as OD being a way of continuing the ancestor’s way of life, the practice of siting latrines far away from home, shared latrines being associated with evil spirits, etc. that motivate the practice of open defecation.

### Study limitation

The study was quantitative. A mixed method with the qualitative component would have offered a deeper understanding and better appreciation of subjects related to the social, cultural, and economic perspective of latrine ownership and utilisation in the study area. Again, this study involved only 20 out of the over 100 communities. A larger sample of the communities would have been ideal for adequate inferences and generalizations.

## Data Availability

Data is provided within the manuscript.
